# Cannabidiol in Food and Food Supplements: Drug, Novel Food and Hazard Triangle

**DOI:** 10.3390/molecules31132287

**Published:** 2026-07-01

**Authors:** Ljilja Torović, Katarina Urumović, Dunja Kobiljski, Branislava Srđenović Čonić

**Affiliations:** 1 Department of Pharmacy, Faculty of Medicine, University of Novi Sad, Hajduk Veljkova 3, 21000 Novi Sad, Serbia; katarina.bijelic@mf.uns.ac.rs (K.U.); branislava.srdjenovic-conic@mf.uns.ac.rs (B.S.Č.); 2 Center for Medical and Pharmaceutical Investigations, Faculty of Medicine, University of Novi Sad, Hajduk Veljkova 3, 21000 Novi Sad, Serbia; 3 Department of Industrial Engineering and Management, Faculty of Technical Sciences, University of Novi Sad, Trg Dositeja Obradovića 6, 21000 Novi Sad, Serbia; dunjakobiljski@uns.ac.rs

**Keywords:** cannabinoids, food, hemp, public health, RASFF, THC

## Abstract

The concept of the “cannabidiol (CBD) Hazard Triangle” reflects the unique position of CBD at the intersection of three overlapping dimensions: CBD as a substance associated with medicinal and pharmacological effects (“Drug”); CBD as a food ingredient subject to the EU Novel Food regulatory framework (“Novel Food”); and CBD as a potential source of food safety concerns (“Hazard”). This study investigates the growing presence of CBD-containing food products, their associated regulatory challenges, safety concerns, and market dynamics through an analysis of notifications reported in the EU Rapid Alert System for Food and Feed (RASFF), complemented by evidence from the scientific literature and authoritative regulatory sources. During the eight years (2018–2025), more than 400 CBD-related notifications were reported, predominantly involving food supplements (66.7%) and confectionery products, particularly gummies (12.6%). Significant discrepancies between the labelled and actual CBD content were frequently identified, along with unauthorized health claims implying therapeutic benefits. CBD-containing products were also found to contain other cannabinoids, most notably tetrahydrocannabinol (THC), which was reported in 26.7% of CBD-related hazard notifications. In several cases, THC concentrations exceeded legally permitted limits. Furthermore, these products are often marketed in forms that may promote casual or unintentional consumption, including by children. Overall, the widespread availability of CBD-containing food products raises important safety and regulatory concerns, particularly for vulnerable population groups. The CBD food market remains highly heterogeneous, characterized by inconsistent labelling practices, strong consumer demand, and increasing regulatory pressure. These findings underscore the need for clearer regulatory frameworks, improved market surveillance, and harmonized standards. Further research is essential to address unresolved issues related to product safety, quality, and market integrity.

## 1. Introduction

**Figure A1:**
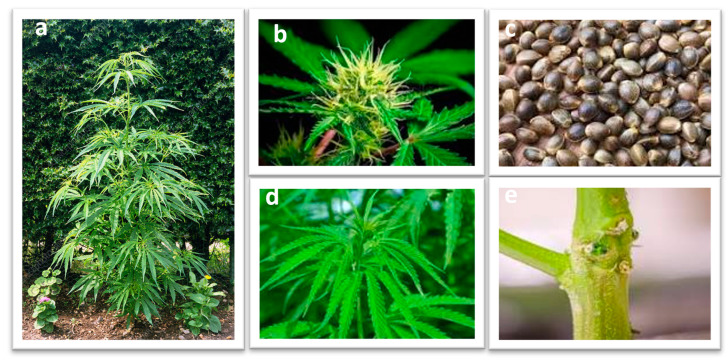
*Cannabis sativa* L. Plant (**a**); inflorescence (**b**); seed (**c**); leaf (**d**); stem (**e**).

*Cannabis sativa* L. is among the oldest cultivated plant species in human history, with applications that have evolved from traditional textile and industrial uses to pharmaceutical, nutraceutical, and food-related purposes [1,2]. It is an annual dioecious flowering plant of the Cannabaceae family, morphologically distinguished by its characteristic palmate compound leaves with serrated leaflets, erect angular stems, and a robust taproot system (Appendix A, Figure A1) that underpins its remarkable adaptability to a wide range of agroclimatic conditions [3,4,5]. The plant’s extensive geographical reach and its long-standing role in the cultural, medicinal, and agricultural traditions of diverse societies across the globe are perhaps best illustrated by the remarkable variety of local names it has accumulated over millennia. In Southern Africa it is known as Dagga (Afrikaans), Mbanje or Imbanje (Shona/Ndebele), Ntsangu or Nsangu (Xhosa/Zulu), and Umya; across South Asia it carries names such as Bhang, Ganja, and Charas in India, Kanchavu in Malayalam-speaking communities, and Gajiimaa in Nepal; in East Asia it is referred to as Sam in Korean and Taima in Japanese, while the term Ganja remains in widespread use throughout Malaysia and Indonesia; in the Americas and Europe it is most commonly encountered as Marijuana or Marihuana, Weed, Pot, Grass, Hemp, Maconha, and Grifa, and across the Middle East and North Africa, Hashish and Al-bhango are among the most enduring designations [3,4,5,6].

The plant produces a diverse array of secondary metabolites, including terpenes, flavonoids, stilbenoids, and phenolic acids, alongside over 100 phytocannabinoids—a class of terpenophenolic compounds unique to the genus—of which the most pharmacologically significant are Δ^9^-tetrahydrocannabinol (THC) and cannabidiol (CBD) (Appendix A, Table A1). These cannabinoids accumulate predominantly in the capitate-stalked glandular trichomes of female flowers, initially as their acidic precursors (THCA and CBDA, respectively) [4,7]. In the contemporary context, a practical distinction is commonly made between drug-type cannabis, characterized by high concentrations of psychoactive cannabinoids, and industrial hemp, primarily based on chemical composition and regulatory status [8,9]. The principal distinction relates to the cannabinoid profile, particularly the content of THCA, which is decarboxylated to THC, the main psychoactive cannabinoid [2,8]. Owing to the regulatory limits imposed on its THC content, hemp is generally considered a lower-risk source of food ingredients and has gained increasing prominence in the food industry through the development of products such as seeds, oils, protein preparations, and functional food ingredients [1,8].

Among hemp-derived phytocannabinoids, CBD has been the most extensively investigated in the context of foods and food supplements. CBD is a non-psychoactive cannabinoid frequently associated with potentially beneficial biological activities, including anti-inflammatory, antioxidant, analgesic, anxiolytic, and neuroprotective effects, which have contributed to its widespread promotion in wellness-oriented products. Consequently, the market for CBD-containing foods and food supplements has expanded rapidly in recent years [1,2,8,9,10].

Despite this expansion, the legal status of CBD within the European Union (EU) remains insufficiently harmonized, resulting in a complex and fragmented regulatory framework that differs among Member States. In January 2019, the European Commission (EC) classified extracts of *Cannabis sativa* L., products containing such extracts as ingredients, extracts of other cannabinoid-containing plants, and synthetically produced cannabinoids as novel foods [11,12]. This classification was based on the conclusion that CBD had not been consumed to a significant extent within the EU before 15 May 1997. Accordingly, CBD products may be placed on the EU food market only following a successful safety assessment and authorization procedure under the Novel Foods Regulation [13], thereby imposing substantial limitations on market access.

At the same time, the EC acknowledged that certain hemp-derived products, including hemp seed oil, hemp seed flour, and defatted hemp seeds, had a documented history of consumption until 1997 and therefore do not require specific authorization [6]. In July 2020, the EC suspended ongoing applications for CBD authorization under the Novel Foods Regulation and informed applicants that CBD products might fall outside the scope of EU food legislation [14]. The EC further suggested that CBD and hemp flower extracts could be more appropriately regulated as narcotic substances under the United Nations Single Convention on Narcotic Drugs [15]. However, in 2021, the European Court of Justice ruled that CBD cannot be classified as a narcotic drug provided that it is extracted from the whole *Cannabis sativa* plant and contains low levels of THC. This judgment represented a significant legal milestone, enabling the free movement of compliant CBD products within the EU internal market. Nevertheless, although the sale of CBD-infused edible products remained restricted [11], various CBD product formats continued to be marketed. More recently, in February and March 2026, the EC terminated several procedures related to the authorization of CBD for placement on the EU market [16].

The European Food Safety Authority (EFSA) has repeatedly emphasized that the safety of CBD as a food ingredient cannot currently be established because of substantial data gaps, particularly regarding hepatotoxicity, drug–drug interactions, reproductive and developmental toxicity, and long-term exposure effects [13,17]. In its most recent assessment, EFSA proposed a provisional safe intake level of approximately 2 mg CBD/day (0.0275 mg/kg bw/day for a 70 kg adult) under highly restrictive conditions, including the use of highly purified CBD, exclusion of nanoformulations, adequately characterized production processes, and the absence of genotoxicity concerns [18]. EFSA also stressed that this value should not be interpreted as a general safety threshold applicable to all CBD-containing products currently available on the market.

An additional challenge in exposure assessment arises from the fact that cannabinoid-related intake cannot be evaluated solely based on declared CBD concentrations. Hemp-derived products may also contain acidic cannabinoid precursors, such as THCA and cannabidiolic acid (CBDA), while technological processing steps, including heating, baking, extraction, and storage, may alter cannabinoid composition through decarboxylation and related chemical transformations [1,2,19]. Furthermore, products marketed as “CBD products” frequently contain additional cannabinoids, including THC, due to natural co-occurrence, use of full-spectrum extracts, incomplete purification procedures, raw material variability, contamination, and technological processing conditions. Labelling inaccuracies and substantial batch-to-batch variability further complicate exposure assessment and official control activities [2,10,19,20]. In general, CBD products are considered legally compliant if THC concentrations remain below 0.2%, although certain countries permit limits of 0.3% or even 0.6%, whereas others apply a zero-tolerance approach. By comparison, Switzerland permits THC concentrations of up to 1% [21].

At present, Epidyolex^®^ (Epidiolex^®^ outside the EU) remains the only oral CBD-based medicinal product authorized in the European Union. It contains highly purified CBD (≥98%) derived from *Cannabis sativa* L. and dissolved in sesame oil. As an orphan drug, it has received regulatory approval following a comprehensive evaluation by the United States Food and Drug Administration, the European Medicines Agency, and the European Commission. The product is indicated for the treatment of severe epilepsy syndromes, including Lennox–Gastaut syndrome, Dravet syndrome, and tuberous sclerosis complex [22]. The authorization of Epidyolex^®^ highlights the distinction between pharmaceutical-grade CBD, whose quality, safety, and efficacy have been formally assessed, and CBD-containing food products, for which significant safety and regulatory uncertainties remain.

Overall, hemp- and CBD-containing food products represent an important public health and regulatory challenge, particularly for vulnerable population groups, including children and adolescents, pregnant and breastfeeding women, older adults, individuals with liver disease, and consumers receiving concomitant pharmacotherapy, owing to differences in susceptibility and the potential for CBD–drug interactions [13,18,23]. Despite unresolved safety concerns and existing regulatory restrictions, CBD-containing foods continue to be marketed and are repeatedly identified during official controls, indicating persistent non-compliance and ongoing challenges for food safety authorities [10,13,22].

The present study examines the EU regulatory landscape, where CBD is regulated as a novel food whose safety has not yet been conclusively demonstrated owing to substantial scientific uncertainties and unresolved data deficiencies [13,17]. The study aimed to systematically analyze notifications recorded in the Rapid Alert System for Food and Feed (RASFF), a publicly accessible centralized database documenting analytical findings and regulatory actions reported by EU Member States [24]. RASFF notifications related to CBD-containing food products were evaluated to identify temporal trends, food categories involved, and enforcement patterns, thereby contributing to a clearer understanding of CBD-related non-compliance within the European food market.

**Figure 1:**
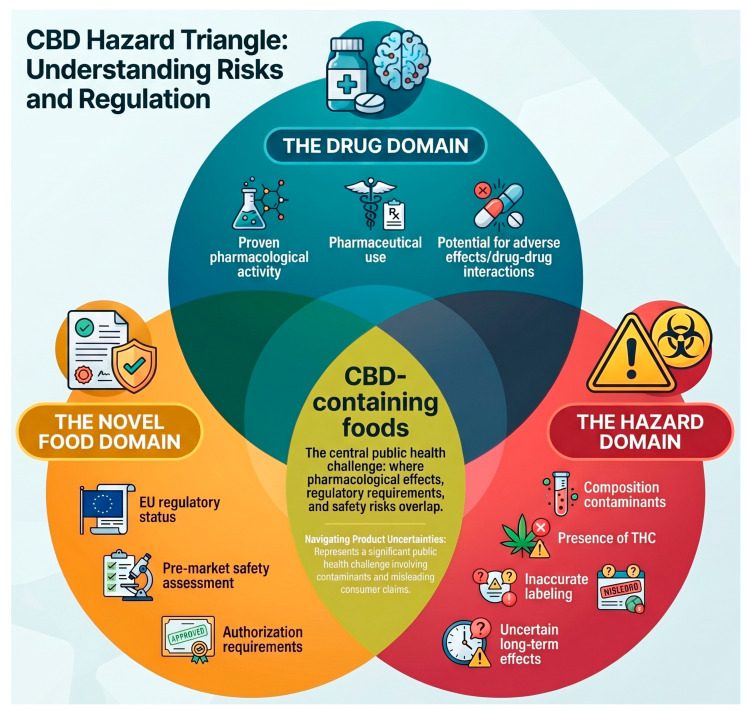
CBD Hazard Triangle. CBD-containing foods occupy the intersection of three domains: Drug (pharmacological activity and potential for interaction), Novel Food (regulatory status requiring authorization and safety assessment), and Hazard (food safety concerns related to composition, contaminants, THC co-occurrence, labelling inaccuracies, and uncertain long-term effects).

The concept of the “CBD Hazard Triangle” (Figure 1) reflects the unique position of CBD at the intersection of three overlapping dimensions: (i) CBD as a substance associated with medicinal and pharmacological effects (“Drug”), (ii) CBD as a food ingredient subject to the EU Novel Food regulatory framework (“Novel Food”), and (iii) CBD as a potential source of food safety concerns (“Hazard”). The Drug pillar refers to the well-documented biological activity of CBD, its use in pharmaceutical products, and the potential for adverse effects and drug–drug interactions. The Novel Food pillar reflects the current EU regulatory status of CBD, which requires pre-market safety assessment and authorization due to the absence of a documented history of significant consumption before 1997. The Hazard pillar encompasses food safety risks associated with CBD-containing products, including uncertainties regarding long-term safety, variability in CBD content, the presence of THC and other cannabinoids, misleading health claims, inaccurate labelling, and the potential for unintended exposure, particularly among vulnerable population groups. Together, these three pillars illustrate why CBD-containing foods and food supplements remain both a regulatory and public health challenge.

## 2. Results and Discussion

### 2.1. RASFF Notification Trends by Years

A total of 427 CBD-related notifications were identified in the RASFF database during the period 2018–2025 (Table 1). Following a marked increase after 2018, the annual number of notifications has remained high since 2019. It is uncertain whether significant spikes in notifications in 2023 and 2025 (77 notifications each) were prompted by targeted, localized intensification of regulatory oversight, but were indisputably related to confectionery and food supplements.

Concerning notification classification and risk decision, informational notifications predominated throughout the observed period. However, beginning in 2023, the category “potential risk” replaced “undecided” as the most frequently assigned risk decision. This shift may be explained by the subsequent introduction of a more precise classification framework and a finer differentiation of risk decision categories within the RASFF system [24].

### 2.2. RASFF Notification Trends by Product Matrices

When food categories were examined, the category “dietetic foods, food supplements, and fortified foods” predominated throughout the study period, except in the final year. Particularly pronounced peaks were observed in 2019 and 2023, with 52 notifications recorded in each year. Overall, this category accounted for 285 notifications, representing 66.7% of all CBD-related notifications identified between 2018 and 2025.

In contrast, notifications related to confectionery products became increasingly prominent from 2023 onwards, reaching 29 notifications in 2025. With a total of 54 notifications (12.6%), confectionery represented the second most frequently reported category. Other categories included “other food products/mixed” (7.2%), cocoa and cocoa preparations, coffee and tea (5.6%), fats and oils (2.6%), and non-alcoholic beverages (2.1%). Additional food categories, including honey and royal jelly, cereals and bakery products, alcoholic beverages, food additives and flavourings, nuts and seeds, and ices and desserts, were reported only sporadically, each accounting for less than 1% of total notifications.

**Figure 2:**
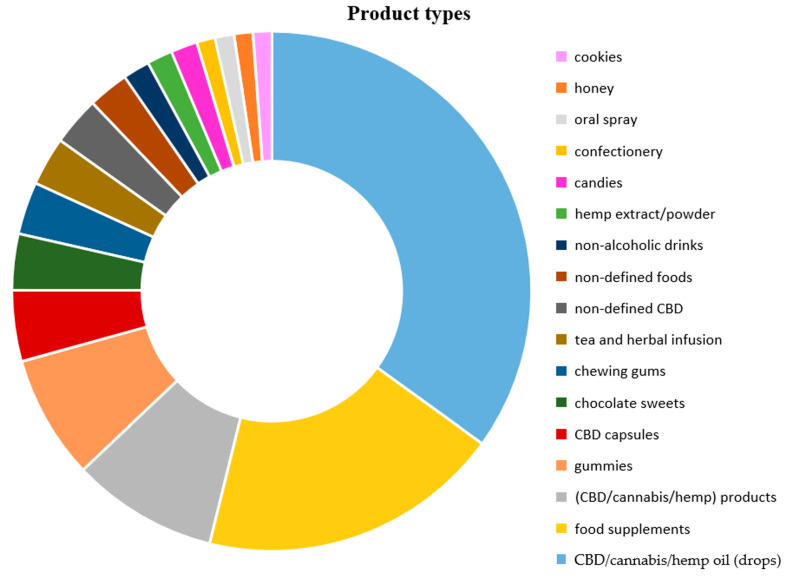
Distribution of CBD-related notifications by product types (≥5 notifications; product types reported in 1 to 4 notifications: biscuits, bonbons, crystals, distillates, ice cream, alcoholic beverages, jellies, lollypops, pasta, syrup, water, and coffee) (RASFF, 2018–2025).

Overall, the findings indicate that CBD-related notifications were predominantly associated with food categories characteristic of supplement-type products (Figure 2, Supplementary Material Table S1), whereas conventional food products were less frequently involved. Most notifications concerning supplement-type products referred to CBD/cannabis/hemp oils (150 notifications), unspecified food supplements (81 notifications), and capsules (19 notifications), reflecting the current market and regulatory landscape. CBD products are primarily marketed in “wellness-oriented” formats, including oils, drops, capsules, and fortified products, rather than as conventional foods. In addition, the investigated dataset included 39 notifications classified only as unspecified “CBD/cannabis/hemp products”, which limited the possibility of obtaining more detailed insight into the specific product types involved.

### 2.3. Manufacturing and Safety Implications of Unit-Dose Edibles

The increasing involvement of confectionery products in recent years is particularly noteworthy, as this category includes unit-dose edible formats such as gummy candies, chewing gums, and lollipops. Of the 54 confectionery-related notifications identified, approximately one-half concerned gummies, making them the most frequently reported confectionery product type. Nevertheless, in the present dataset, the product-level classification appeared to be more informative than the broader food-category classification. Specifically, a total of 33 notifications were related to gummies, the majority of which were categorized as confectionery products, whereas a smaller number were classified under dietetic foods, food supplements, fortified foods, or mixed food products. This variability reflects differences in the interpretation of the intended purpose and regulatory classification of such products.

Gummy-based products present specific technological and safety challenges related to dose uniformity. Achieving homogeneous cannabinoid distribution within gelatin- or pectin-based matrices requires strict process control, and insufficient homogenization may result in considerable variability both between batches and among individual units. Such variability compromises labelling accuracy and increases the risk of unintended overexposure, particularly because these products are frequently consumed casually as snack-like items [10,25].

These concerns may be further exacerbated by product presentation and marketing practices. In some cases, edible CBD products are packaged and labelled in a manner resembling conventional confectionery products, often accompanied by “natural” or “wellness-oriented” terminology. Such presentation may contribute to an underestimation of potential risks and increase the likelihood of inappropriate use or accidental ingestion, particularly among children [26,27]. Published reports describing pediatric exposures associated with edible cannabis products further support these concerns [23,26].

### 2.4. Global Literature Synthesis of CBD-Content Variance and Label Discrepancies

To contextualize the RASFF findings, Table 2 summarizes studies published since 2020 that reported CBD concentrations in hemp- and cannabis-based foods and related oral consumer products outside the RASFF framework. Although the reported concentrations were presented according to the methodologies and units used in the original publications, thereby limiting direct numerical comparisons across product matrices, the collected data provide a broad overview of CBD concentration ranges and labelling compliance.

Notably, several studies identified products marketed as CBD-containing that contained no detectable CBD [31]. Similarly, all hemp seed oils analyzed in a Hungarian study [28], as well as several hemp seed oils reported in other studies [36,37,41], were CBD-free. The absence of detectable CBD was also reported in certain hemp seed samples [41], hemp protein products [37], confectionery items [10,37], tea plant material [10], and beverages [31].

Substantial deviations between labelled and analytically determined CBD concentrations were frequently reported. For example, an analysis of food supplements marketed in Hungary demonstrated that only 2 of 12 products were correctly labelled, whereas 2 were underlabelled and 8 overlabelled [28]. Similar inconsistencies were observed in studies examining CBD oils. The proportion of accurately labelled products (defined as ±10% deviation from the declared concentration) ranged from 3/8 to 10/23 analyzed samples [10,30,32,33,35]. Underlabelled products were also frequently reported [10,32,33], as were overlabelled products [10,31,32,33]. In addition, several products lacked any explicit CBD concentration claim on the label despite being marketed as CBD-containing products [30,31,33]. Among confectionery products analyzed in a German study, three products were appropriately labelled, one was overlabelled, and four lacked a specific CBD label claim [10]. Beverages, including both alcoholic and non-alcoholic products, also exhibited marked inconsistencies, with only a limited number of products appropriately labelled, while many were underlabelled, overlabelled, or lacked CBD declarations entirely [10,33]. Some studies reported extremely large deviations from declared CBD concentrations, reaching up to 226% [45]. Furthermore, a Portuguese study identified products that did not explicitly declare the presence of *Cannabis sativa* extracts, despite extensive use of cannabis-related imagery and terminology on the packaging [31].

Another important labelling issue with significant implications for consumer purchasing decisions concerns the use of health claims. A Hungarian study simulating online consumer purchasing behavior demonstrated that, among 16 online retailers selling hemp-derived products and CBD oils, two retailers displayed direct health claims on product pages, whereas ten indirectly promoted potential health benefits elsewhere on their websites [32]. Although EU regulations prohibit attributing therapeutic properties to food supplements, and no authorized or pending health claims currently exist for CBD, content analysis identified approximately 30 different purported medical “indications” presented by retailers. The most frequently referenced conditions included autism spectrum disorders, anxiety, depression, panic attacks, bipolar disorder, and cardiovascular-related conditions such as hypertension, arrhythmia, and hypercholesterolemia [37]. Other studies have similarly reported frequent violations of regulatory requirements related to health claims and product promotion [46,47]. Concerns regarding misleading health-related promotion extend to social media platforms. An analysis of medical claims related to CBD products on Twitter identified pain management, anxiety reduction, sleep improvement, and stress relief as the four most commonly promoted “therapeutic” applications [48].

Particular considerations also apply to products intended for the preparation of infusions, such as coffee and tea plant materials, where the transfer rate of cannabinoids from the solid matrix into the beverage represents an important factor in exposure assessment. According to the precautionary principle, a maximal transfer rate of 100% is generally assumed when estimating potential cannabinoid exposure [49]. However, recent studies suggest that this assumption may substantially overestimate actual exposure, as the transfer of cannabinoids into tea infusions appears to be relatively limited under typical preparation conditions [50].

### 2.5. CBD Co-Reporting with THC and Other Cannabinoids

**Figure 3:**
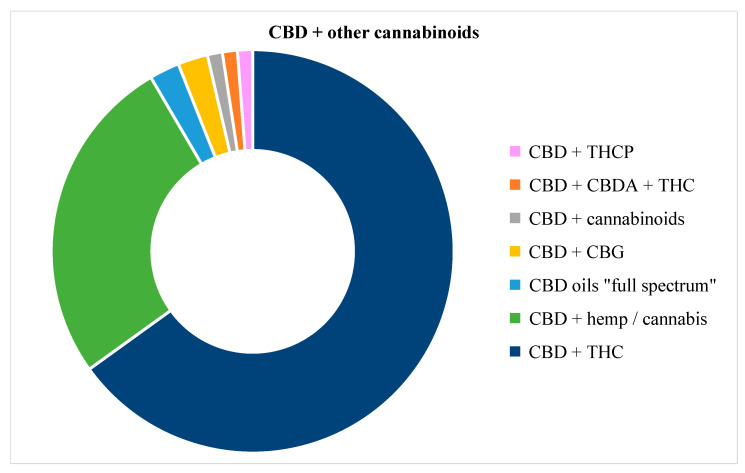
Distribution of CBD co-reporting with THC and other cannabinoids (Note: additional binary combinations of CBD with 10-OH-HHC and CBN, ternary with CBN + CBC, THC + HHC, and H_4_CBD + THC, and quinary with H_4_CBD + THCV + THCP + THC, as well as THC + hemp extract were observed once each) (RASFF, 2018–2025).

Although the present study primarily focused on CBD, the identified compliance issues and potential risks were not limited exclusively to this cannabinoid. Given the frequent co-reporting of CBD with other cannabinoids—sometimes specifically identified and in other cases reported more generally as cannabinoids or hemp/cannabis-related substances (Figure 3, Table S2)—the potential for consumer misperception and unintended exposure was considered highly relevant. Such findings further emphasize the complexity of cannabinoid-containing products and the challenges associated with accurate characterization, labelling, and exposure assessment.

Foods marketed as CBD-containing products are frequently not chemically selective and may contain detectable, but undeclared, amounts of THC. Among notifications retrieved using CBD as the primary reported hazard, THC was additionally reported in 114 cases, corresponding to a co-occurrence rate of 26.7%. Of these, 108 notifications involved an exclusive CBD–THC combination, whereas 6 also included other cannabinoids, indicating the frequent simultaneous presence of this psychoactive cannabinoid alongside CBD [2,19,20,42].

In this context, tetrahydrocannabinolic acid (THCA) is of particular relevance because it may be converted into psychoactive THC through decarboxylation during processing procedures involving heating, such as baking or cooking, as well as potentially during storage-related changes in cannabinoid composition over time [2,25]. Consequently, consumer exposure risk cannot be estimated solely from declared CBD content, and products marketed as CBD-containing cannot automatically be considered free of risks associated with psychoactive cannabinoids [14,37].

Moreover, 44 notifications referring to CBD in hemp/*Cannabis sativa* products, four notifications involving “full-spectrum” CBD oils, and two notifications concerning consignments containing CBD together with other cannabinoids may also have involved psychoactive cannabinoids, potentially increasing the estimated co-occurrence of THC-related substances with CBD products to 38.4%. Importantly, 33 out of the 114 THC-related notifications specifically identified the presence of elevated or unsafe THC concentrations in CBD products as the sole reported hazard. In such cases, THC was categorized as a biological hazard, whereas CBD itself was classified as an unauthorized novel food ingredient. An earlier review of RASFF notifications concerning Δ^9^-THC in CBD oils and food supplements between 2020 and 2022 identified 61 comparable reports [51]. Similarly, review articles have reported that Δ^9^-THC is relatively common in CBD oil preparations, typically at concentrations of 0.1−0.3% [45].

Findings reported outside the RASFF system further corroborate these observations (Table 2). In contrast to food supplements analyzed in Hungary, where THC was not detected [28], 98 out of 131 supplements examined in German market surveillance studies tested positive for THC, with concentrations reaching up to 3400 mg/kg [29]. Regarding CBD oils, THC concentrations below the applicable EU legal threshold were reported in 6/23 [30], 6/11 [33], and 52/80 samples [34]. Similar findings were observed for hemp seed oils, where THC was detected in 19/20 [38], 8/43 [39], and 102/137 samples [29], as well as in hemp seeds, where 89 out of 93 analyzed samples contained detectable THC [29]. The occurrence of THC was also reported in a wide range of additional hemp-derived food products, including all analyzed hemp flour samples (7/7), 12/33 pasta samples, 12/22 bakery products [19], 78/89 hemp tea products [29], most infusion products among 129 tested samples, most tea plant material samples among 100 tested samples, and the majority of analyzed coffee samples, including 6/9 [19] and the majority of the 172 samples in another study [20]. Regulatory THC limits were exceeded in several product categories, including hemp seed oils [40,41,43], hemp seeds [39,43], and hemp flours [39]. Furthermore, because some countries apply a “zero tolerance” policy for Δ^9^-THC, all but one hemp seed oil sample and all analyzed hemp seed samples investigated in Türkiye were considered legally non-compliant [38]. Thereby, a lack of harmonized minimum reporting limits (MRLs) and analytical cut-offs across the European perimeter creates massive administrative disruptions for cross-border e-commerce and trade.

Studies evaluating THC exposure have suggested that adverse effects associated with some commercial CBD products may primarily result from THC contamination rather than from CBD itself. For example, among 293 analyzed CBD oils, 28 products were associated with Δ^9^-THC exposure above the lowest observed adverse effect level (LOAEL; 2.5 mg/day), 131 resulted in exposure between the acute reference dose (ARfD) and LOAEL, and only 134 remained below the ARfD [22]. Similarly, consumption of the recommended serving size (14 g) of three out of eight THC-positive hemp seed oils would result in ARfD exceedance for a 70 kg adult [29]. In a study investigating the effects of commercially available edible cannabinoid products, participants consumed THC-dominant, CBD-dominant, or combined THC + CBD products. Participants exposed to combined THC + CBD products reported positive effects such as intoxication, elation, and product liking, as well as psychotomimetic effects including paranoia and tension, at levels comparable to those observed in participants consuming THC-dominant products, despite lower plasma THC concentrations [52].

An additional issue concerns the labelling of THC content, particularly the use of “THC-free” claims. In a United States study, 5 out of 21 CBD oils marketed as “THC-free” contained detectable Δ^9^-THC concentrations ranging from 0.015 to 0.656 mg/mL [34]. Similarly, ten hemp seed oils available on the EU market contained between 0.44 and 13 mg/kg THC equivalents, whereas only one out of ten hemp seed samples and none of the three hemp flour samples analyzed could genuinely be classified as “THC-free” [29]. Importantly, implementation of EU regulations establishing maximum limits for Δ^9^-THC [53] has reportedly contributed to a decline in THC concentrations observed in products sampled after 2023.

Within the present RASFF dataset, one notification described an adverse effect associated with the use of 5% CBD oil, while another reported intoxication following the consumption of gummies containing THC and H4CBD in 2023 and 2024, respectively. Nevertheless, it should be emphasized that published cases of pediatric poisoning have predominantly been associated with THC-containing edible cannabis products rather than products marketed exclusively as CBD formulations. A recent review reported that Canadian provinces permitting edible cannabis products experienced a substantial increase in pediatric poisonings related to unintentional consumption, with hospitalizations occurring predominantly among children aged 3–4 years [23]. The same review cited data from Colorado demonstrating that pediatric hospital visits doubled and poison control centre calls increased by approximately 50%, with a median age of 3 years among affected children. Importantly, Canadian data indicated an increase in pediatric poisoning rates from 57.42 to 318.04 per 1000 when comparing periods before and after legalization in exposed areas [23]. The review further emphasized that products such as chocolates, gummies, and baked goods may pose particular risks due to their close resemblance to conventional confectionery, thereby increasing the likelihood of accidental ingestion by children [23]. Concurrently, market surveillance studies of CBD and hemp-derived products have repeatedly demonstrated the presence of THC in products marketed as CBD-dominant or even “THC-free”, indicating that confectionery-like cannabinoid products may represent a broader cannabinoid exposure concern rather than an exclusively CBD-related issue [54].

### 2.6. CBD-Related Notifications by Notification Classification and Risk Decision

**Figure 4:**
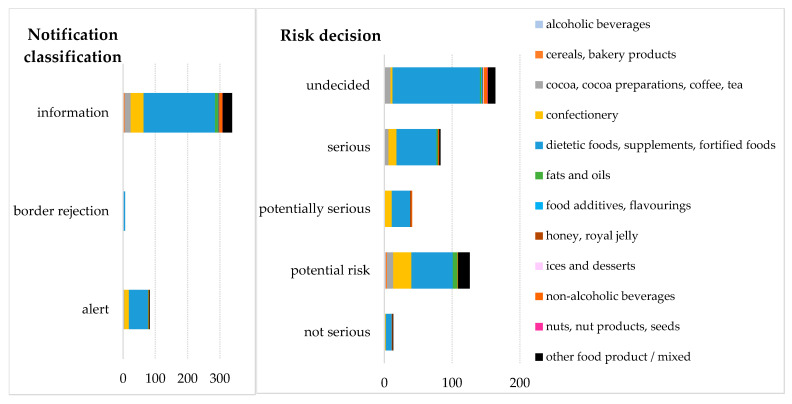
Distribution of CBD-related notifications by notification classification and risk decision across food categories (RASFF, 2018–2025).

The distribution of CBD-related notifications by RASFF notification classification and risk decision is presented in Figure 4 (Tables S3 and S4).

The majority of notifications were classified as information (79.2%), while alerts accounted for 19.4% of cases, and border rejections constituted only 1.4% of the total dataset. This pattern was driven primarily by the category “dietetic foods, food supplements, and fortified foods”, which represented the dominant category in both informational (65.4%) and alert notifications (71.1%). In the context of CBD-containing products, the predominance of informational notifications is consistent with the fact that a substantial proportion of non-compliance within the EU is associated with the novel food status of CBD and related procedural or legal irregularities, including the use of unauthorized ingredients. Such cases may require information exchange and follow-up measures even when an immediate health risk is not explicitly identified at the time of reporting. At the same time, the considerable proportion of alert notifications indicates that many non-compliant products had already entered the market and therefore required rapid intervention by competent authorities.

It should be noted that the provisional safe intake level for CBD (~2 mg/day) was introduced only in the 2026 EFSA update, whereas the RASFF notifications analyzed in the present study covered the period from 2018 to 2025. Consequently, most notifications were generated within a regulatory context in which CBD safety was primarily characterized by scientific uncertainties and significant data gaps rather than by an established quantitative reference dose [13,17,18].

Furthermore, the high proportion of both informational and alert notifications within the category of dietetic foods, food supplements, and fortified foods supports the interpretation that regulatory attention has been directed particularly toward product formats intended for repeated consumption and marketed for “wellness-oriented” purposes. Such products are commonly consumed on a daily basis and may therefore contribute to higher cumulative exposure, which is relevant not only in the context of hepatotoxicity but also regarding the potential for interactions with concomitant medications, repeatedly emphasized by EFSA [13]. Collectively, these findings suggest that the observed notification pattern reflects both the specific legal status of CBD within the EU and the practical difficulties associated with controlling a rapidly expanding and highly heterogeneous market characterized by inconsistent labelling practices and variable product quality [1,10].

Regarding risk decisions, the largest proportion of notifications was classified as “undecided” (38.4%), followed by “potential risk” (29.0%) and “serious risk” (19.4%). Smaller proportions were categorized as “potentially serious” (9.6%) and “not serious” (3.0%), whereas no notifications were classified as “no risk”. The predominance of “undecided” (78.6%) and “serious risk” (71.1%) classifications within the category of dietetic foods, food supplements, and fortified foods further indicates that overall risk-decision patterns were largely driven by the dominance of this product segment in the dataset, rather than by notifications concerning conventional food products. The substantial proportion of “undecided” notifications suggests that, in many CBD-related cases, a definitive risk assessment could not be established at the time of reporting. This finding is consistent with the broader regulatory context during the study period, in which CBD-related non-compliance was frequently identified, while scientific uncertainties and insufficient toxicological data limited the ability to assign a clear risk level [13]. In practice, the “undecided” classification likely reflects situations in which the presence of regulatory non-compliance was evident, but the available scientific evidence was insufficient to support a confident assessment of risk severity within the RASFF framework. Nevertheless, this places the substantial practical burden on enforcement. When national authorities log an alert as “undecided”, it leaves food operators and border inspectors in a legal limbo regarding whether a product must be seized under acute safety laws or restricted procedurally. At the same time, the notable proportion of notifications classified as “serious risk” indicates that a considerable number of cases were regarded as sufficiently significant to warrant heightened regulatory attention and rapid control measures.

### 2.7. CBD-Related Notifications by Origin and Notifying Countries

**Figure 5:**
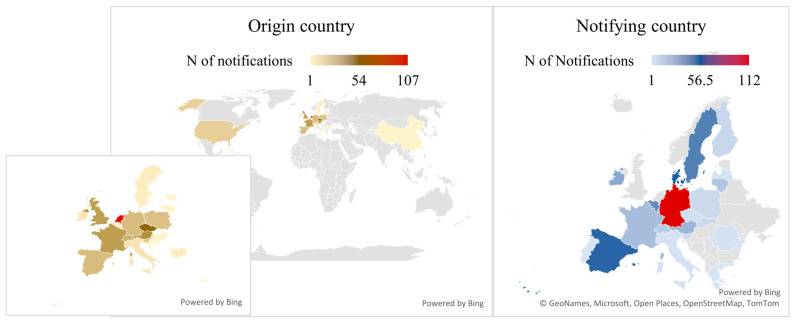
Geographical distribution of CBD-related notifications by country of origin and notifying country (RASFF, 2018–2025) (Map generated using Microsoft^®^ Excel^®^ Powered by Bing™. Contains data from GeoNames, Microsoft, Open Places, and OpenStreetMap).

The geographical distribution of CBD-related RASFF notifications by country of origin and notifying country is shown in Figure 5.

The Netherlands was the most frequently reported country of origin, accounting for 107 notifications, followed by Czechia (52), Austria (37), and both France and the United Kingdom (32 each). A substantial number of notifications were also associated with Switzerland (26), Spain (21), and Germany (21). Outside Europe, 14 notifications identified the United States as the country of origin, whereas China and Hong Kong were reported only once each. It should be noted that the total number of origin-country entries did not fully correspond to the total number of notifications (n = 427). In several cases, more than one country of origin was reported within a single notification, resulting in minor discrepancies when data were summarized according to origin. Furthermore, 30 notifications were recorded as having an unknown country of origin, thereby limiting the precision of geographical interpretation. This lack of primary supply-chain traceability constitutes an independent risk vector, making targeted batch recalls nearly impossible during a contamination event.

The reported country of origin should be interpreted as the origin information provided within the RASFF notification itself, rather than as a direct indication of the actual site of cannabinoid production or extraction. CBD-containing products are frequently derived from extracts distributed through complex international supply chains, and the “origin” reported in notifications may therefore reflect only the provenance information available to authorities at the time of official control rather than a clearly defined manufacturing location. Against this background, the predominance of European countries among origin entries likely reflects the importance of European distribution channels and intra-European trade within the CBD market, whereas only a relatively small proportion of notifications explicitly referred to non-European origins [2,10,19]. An additional important observation concerns the occurrence of notifications reporting unknown or multiple countries of origin. Such findings indicate limitations in traceability and supply-chain documentation for CBD-containing products. In the context of risk management, reduced traceability is not merely a matter of incomplete data quality but may directly affect the timeliness of enforcement actions, the complexity of targeted controls, and the effectiveness of subsequent regulatory follow-up measures [10].

Furthermore, origin patterns should be interpreted together with the frequently reported variability in cannabinoid composition and labelling quality. Market surveillance studies have repeatedly demonstrated that products marketed as CBD-containing may also contain quantifiable concentrations of THC or THCA, accompanied by substantial discrepancies between labelled and analytically determined cannabinoid concentrations (Table 2). These findings suggest that non-compliance is not restricted solely to the regulatory status of the products but may also reflect variability in raw material quality, manufacturing practices, extraction procedures, and overall process control [10,19,20,42]. Accordingly, the concentration of notifications associated with particular countries should not be interpreted as a direct indicator of manufacturing standards or production quality within those jurisdictions.

Products marketed through online sales channels appear to represent a particularly challenging segment of the market. In the present dataset, 57 notifications were associated with web shops, highlighting the growing importance of e-commerce in the distribution of CBD-containing products and the additional regulatory challenges. The globalized nature of online retailing poses substantial jurisdictional and enforcement challenges. In particular, conventional interventions such as physical product seizures are often ineffective when products are supplied by third-country online vendors and delivered directly to consumers in small, unit-dose consignments through postal and courier networks. With respect to notifying countries, Germany was the leading notifier, accounting for 26.6% of all notifications, followed by Denmark and Spain, which contributed 12.6% and 11.7% of notifications, respectively. Additional notable contributions were reported by Sweden (9.4%), Belgium (8.2%), and Ireland (5.9%), whereas the remaining 15 countries each accounted for only a relatively small proportion of notifications (0.2–4.4%). Overall, these findings indicate that CBD-related notifications were reported predominantly by a limited number of countries, with Germany clearly representing the principal notifying country within the analyzed dataset. Germany’s strong contribution is consistent with broader RASFF reporting patterns, as the country is regularly listed among the leading notifying Member States in annual EU food safety reports. In addition, the German Federal Office of Consumer Protection and Food Safety plays a central role in the national alert and information framework and in RASFF communications, thereby supporting a high level of reporting capacity and surveillance activity [24].

Importantly, notifying-country patterns should not be interpreted as direct indicators of manufacturing location. Rather, they should be viewed as reflecting jurisdictions in which CBD-related non-compliance is most frequently detected and reported through official control activities. Several factors, including the intensity of surveillance programs, analytical capacity, national enforcement priorities, and the role of specific countries as key distribution nodes within the EU market, influence these patterns. The predominance of a relatively small number of notifying countries, therefore, suggests that CBD-related non-compliance is primarily identified through concentrated surveillance activities conducted in strategically important market jurisdictions.

### 2.8. Consumer Considerations and Implications

In 2021, Malta became the first EU Member State to legalize personal cannabis use and cultivation, while Bulgaria was the first EU country to permit the free sale of hemp-derived CBD products [21]. Developments in this rapidly evolving area have contributed to broader political and cultural normalization of cannabis- and CBD-related products, potentially facilitating market expansion and further emphasizing the need for standardized formulations and additional clinical and toxicological research. Although the medical use of CBD in the form of pharmaceutical preparations is generally recommended only after conventional therapeutic options have failed, the effectiveness of the fragmented European regulatory framework in limiting the non-medical use of CBD-infused products remains questionable.

Currently, systematic information regarding the diversity of CBD products available on the European market, the characteristics of consumers using these products, and the extent of consumption remains limited. Consequently, key research questions concern consumer awareness, satisfaction, product types consumed, and purchasing channels.

The most recent EFSA assessment introduced a provisional safe intake level of approximately 2 mg of CBD/day for adults under restrictive conditions, including the use of highly purified CBD, the exclusion of nanoformulations, and the absence of genotoxicity concerns [18]. The difference between non-nano and nano-formulated products matters because regular CBD taken orally is absorbed poorly, with reported bioavailability of only about 6–9%. By contrast, systems such as nanoemulsions, liposomes, polymer nanoparticles, and solid lipid carriers are made to improve CBD solubility, stability, and absorption [55,56]. EFSA has published wider guidance and reporting tools for assessing risks from nanotechnologies and small particles in the food and feed chain, but these are not specific to CBD. They also do not provide data on how common nano-formulated CBD products are, what they contain, or how much they may add to consumer exposure [57,58].

In this RASFF-based analysis, product descriptions did not include detailed information on formulation. Because of this, it was not possible to reliably separate standard CBD products from nano-formulated or water-dispersible products. This points to an important gap in regulatory oversight. If a product increases CBD absorption, the same labelled dose could lead to higher CBD levels in the body. This may change how the EFSA provisional safe dose should be understood. More generally, when this low provisional value is compared with CBD levels found in commercial oils and supplement-type products, repeated daily use may lead to intakes above this cautious limit [13]. The toxicological basis for these concerns is primarily linked to hepatotoxicity, which remains the critical endpoint identified in EFSA evaluations. Available evidence suggests that CBD exposure may induce elevations in liver enzymes, including alanine aminotransferase (ALT), aspartate aminotransferase (AST), alkaline phosphatase (ALP), and gamma-glutamyl transferase (GGT), and may be associated with liver injury at higher exposure levels, particularly during prolonged use or in susceptible individuals [13,18,23]. In addition to hepatic effects, EFSA has highlighted potential adverse effects involving the gastrointestinal system, including diarrhea, endocrine-related effects, reproductive and developmental toxicity concerns, neurodevelopmental findings observed in prenatal exposure models, and a considerable potential for cytochrome P450 (CYP)- and uridine 5′-diphospho-glucuronosyltransferase (UGT)-mediated drug–drug interactions [13,17,18]. These issues are especially relevant in the context of foods and food supplements, where product use extends beyond medically supervised patient populations and may involve vulnerable consumer groups.

Accordingly, the public health relevance of the present findings is particularly important for vulnerable populations, including children and adolescents, pregnant and breastfeeding women, older adults, individuals with liver disease, and consumers receiving concomitant pharmacotherapy, owing to differences in susceptibility and the potential for CBD–drug interactions. Collectively, the concentration of notifications in supplement-type products and the increasing involvement of confectionery products, particularly gummies, suggest that CBD-related non-compliance is closely associated with product formats intended for frequent and convenient consumption. As briefly mentioned in Section 2.3, the technological challenge of infusing lipophilic cannabinoid extracts into hydrophilic gel matrices (pectin/gelatin) poses a risk of poor homogenization, leading to batch variation and “hot spots”, directly contributing to pediatric exposure risks. At the same time, co-occurring THC/THCA, labelling inconsistencies, and dose non-uniformity remain central challenges for risk assessment, risk management, and regulatory enforcement [10,13,18].

Inter-individual variability should also be considered when interpreting the risks associated with cannabis-derived food products. Recent evidence indicates that cannabinoid responses depend on multiple factors, including route of exposure, food matrix, age, sex, genetic background, and interactions with food components or concomitant medications, making the establishment of universally applicable safe intake levels particularly challenging [59].

The observed notification patterns may also be interpreted in relation to consumer demand and consumer perceptions. Survey-based studies indicate that consumers frequently use CBD products for stress reduction, relaxation, and sleep improvement, despite often possessing limited knowledge regarding dosing, legal status, and the distinction between food supplements and medicinal products. According to a survey conducted among young adults in the United States, the most frequently reported reasons for CBD consumption were stress reduction (65.4%), relaxation (54.8%), and improvement of sleep quality (42.2%). Most participants also reported experiencing at least one adverse effect associated with CBD use, while the majority determined product dosage based primarily on personal estimation or subjective feeling rather than formal guidance [60]. Similarly, a Canadian survey demonstrated that willingness to consume cannabis-infused foods coexisted with concerns regarding overconsumption and risks to children [61]. An Indian study investigating consumer awareness and willingness to pay for hemp-based foods reported that respondents with academic backgrounds in food science and related disciplines demonstrated greater awareness of the distinction between hemp and marijuana. Participants also expressed willingness to consume hemp-based staple foods, snacks, and fast-food products, with health considerations representing the primary motivating factor [62]. A recent cross-sectional study conducted in Portugal provided additional important insights, including high levels of satisfaction among consumers of cannabis-based products, limited ability to distinguish between food supplements and medicinal products, low awareness that cannabinoids and cannabis plant components are not authorized for use in foods, substantial influence of media on product selection, preference for online purchasing channels, and the recommendation of such products by health professionals [63]. The authors emphasized the importance of promoting informed and conscious consumer choices and proposed a Community Knowledge framework for food supplements [63].

Another important issue concerns whether consumers are aware that the amount of cannabinoids present in marketed products does not necessarily correspond to the amount ultimately ingested, owing to the instability of certain cannabinoids during storage and technological processing. This issue is particularly relevant for products intended for heating, cooking, or baking. For example, a study investigating the effects of thermal processing on cannabinoid profiles in hemp seed edible oils demonstrated that exposure to high temperatures (200 °C for 60 min) resulted in a 38% decrease in total cannabinoid concentrations and a 99% reduction in cannabinoid acids, while simultaneously increasing Δ^9^-THC concentrations by 22% [40]. In practice, this means that a consumer who buys a legal, free- or low-THC ingredient for home baking could accidentally cook themselves an intoxicating dose of (newly introduced) narcotic hazard.

Additional concerns relate to unintended cannabinoid-positive findings in biological specimens following the consumption of CBD or hemp-derived oils, particularly in the contexts of anti-doping control, occupational testing, and road safety. A recent systematic review evaluating the occurrence and underlying causes of cannabinoid-positive drug tests emphasized that five of eight evaluated studies concluded that the use of CBD or hemp oils would not result in THC concentrations exceeding standard cut-off thresholds. Nevertheless, trace THC levels close to analytical detection limits were identified in several cases. Furthermore, two out of four studies investigating the potential in vivo conversion of CBD to THC reported no evidence of biotransformation following consumption of CBD or hemp oils [54].

Travel-related issues concerning CBD products also remain important. Although traveling with CBD products is generally permitted between countries where such products are legal, provided THC concentrations remain below 0.3% (*w*/*w*), the fragmented regulatory landscape requires consumers to verify the specific legislation applicable in each destination country. Certain countries, including Sweden and Norway, apply strict non-detectable THC requirements, whereas others prohibit all CBD products regardless of THC concentration [21].

Overall, the CBD food and supplement sector is characterized by substantial heterogeneity, encompassing diverse product formats, ambiguous “hemp/CBD” terminology, and inconsistent dose reporting practices. These factors complicate official control activities, product traceability, and exposure assessment, and likely contribute to the product recalls and RASFF notifications observed in the present study. At the same time, increasing consumer demand and perceived health benefits—often described within the context of a “green rush”—have transformed the sector into a rapidly expanding and commercially intensive market, thereby placing considerable pressure on existing regulatory oversight systems [1,10,64].

Consequently, there is a clear need for additional regulatory guidance regarding analytical methodologies, product characterization, labelling practices, and risk communication within the CBD food and supplement sector. In parallel with improved unit-dose communication, the implementation of child-resistant packaging has also been proposed as an important risk-management measure [23].

## 3. Materials and Methods

Data for this study were retrieved from the Rapid Alert System for Food and Feed (RASFF), a publicly accessible European Union database designed to facilitate the exchange of information regarding food and feed safety risks among the EU Member States. A targeted search was conducted using the keywords “CBD” and “cannabidiol” in the subject field to identify notifications concerning CBD-containing food products. Veterinary/pet food/supplements containing CBD were systematically filtered out by excluding “feed” from the RASFF categories search. Products other than food that can also contribute to CBD exposure in humans, such as cosmetics, topicals, or electronic cigarette liquids, are out of scope of the RASFF and thus a priori excluded from the search. The search covered the period from 1 January 2018 to 31 December 2025, resulting in a total of 427 retrieved notifications.

Following data collection, the dataset was exported and processed in Microsoft Excel (Microsoft Excel v16.0; Microsoft Corporation: Redmond, WA, USA), where notifications were reviewed, organized, and prepared for descriptive analysis. For each notification, the following variables were recorded: reference number, notification date, notification subject, food category, product description, reported hazard, notification classification, risk decision, country of origin, and notifying country. These variables were subsequently used to characterize notifications from both regulatory and food safety perspectives.

Certain notifications included multiple products and/or multiple reported countries of origin. Consequently, minor discrepancies in the total number of observations could occur depending on the variable analyzed. Therefore, the number of observations was interpreted in relation to the specific parameter under consideration.

The analysis focused on: (1) temporal trends in the number of notifications; (2) the distribution of notifications according to food categories and product types; (3) notification classification and associated risk decisions; and (4) the geographical distribution of notifications according to the country of origin and the notifying country. The country of origin was used to identify the reported source of the products, whereas the notifying country was considered relevant for identifying jurisdictions in which such products were most frequently detected and subjected to official controls.

The present study specifically focused on notifications retrieved based on a single reported hazard, namely CBD, as well as additional cannabinoid-related information within notifications, particularly the presence of THC where applicable. The study employed a retrospective descriptive design, and the findings were expressed as absolute numbers and percentages.

To complement the primary CBD-related analysis, a second RASFF dataset was generated for the same period using the search terms “cannabis” and “hemp”. Notifications containing “CBD” and/or “cannabidiol” were subsequently manually screened in Microsoft Excel and removed to avoid duplication with the primary dataset. After this filtering step, 73 additional notifications remained and were analyzed using the same set of variables. These notifications were not included in the original analysis because they cannot be unequivocally linked to CBD. As CBD is not mentioned in any of the notifications included in the secondary dataset, its presence cannot be confirmed. Therefore, the results derived from this dataset are presented separately in Appendix B. It is of interest to note that THC was identified as the underlying cause in 42 of the 73 notifications.

The geographical distribution of the data was visualized using the map feature in Microsoft Excel (Microsoft Excel v16.0; Microsoft Corporation: Redmond, WA, USA), which utilizes the Bing Maps service [65] to aggregate spatial data from OpenStreetMap [66] and GeoNames [67].

## 4. Conclusions

This study systematically analyzed CBD-related RASFF notifications reported between 2018 and 2025, providing an overview of temporal trends, affected food categories, and regulatory characteristics of CBD-related non-compliance within the European food market. Notifications were predominantly associated with dietetic foods, food supplements, and fortified foods, followed by confectionery products, indicating that CBD-related cases primarily involve “wellness-oriented” and unit-dose edible formats rather than conventional foods.

The predominance of “undecided” risk classifications until 2023 reflects persistent uncertainty in CBD risk characterization during the reporting period and suggests that precautionary approaches frequently guided regulatory decision-making. From 2023 onwards, “potential risk” became the leading risk category, accompanied by a substantial proportion of “serious risk” notifications, highlighting the ongoing challenges of risk assessment in a regulatory environment characterized by incomplete toxicological evidence and evolving scientific understanding. Although introduced under restrictive conditions, the EFSA 2026 provisional safe intake level for CBD may facilitate future risk characterization, serving as an immediate turning point that will resolve the pre-2026 “undecided” data bottlenecks highlighted in the current study, and contribute to greater consistency in regulatory assessment.

Importantly, THC was co-reported in a considerable proportion of notifications, emphasizing that market control of CBD-containing products frequently involves broader cannabinoid profiles and the potential for unintended exposure to psychoactive cannabinoids. These findings further underline the importance of considering cannabinoid co-occurrence, cannabinoid transformation during processing, and variability in product composition when evaluating consumer exposure and product safety.

Evaluation of countries of origin revealed a predominance of European supply chains among notified products, while the substantial proportion of notifications involving unknown or multiple origins highlighted persistent shortcomings in supply-chain traceability and product transparency. At the same time, the distribution of notifying countries demonstrated that most notifications originated from a relatively limited number of EU Member States, suggesting that CBD-related non-compliance is primarily identified through concentrated surveillance and official control activities in key market jurisdictions.

Overall, the findings underscore significant regulatory, analytical, and public health challenges associated with the rapidly expanding CBD food and supplement sector. Inconsistent labelling, variable cannabinoid composition, traceability limitations, and the presence of THC in products marketed as CBD-containing collectively emphasize the need for improved quality control, harmonized regulatory approaches, and clearer risk communication as scientific evidence regarding CBD safety continues to evolve.

An important limitation of the current study is that RASFF, as a data source, relies solely on voluntary or reactive reporting by individual EU Member States; therefore, the trends captured primarily reflect active enforcement capacity rather than an exhaustive map of every non-compliant product circulating across the entire European gray market.

The findings highlight the need for harmonized, science-based international action involving regulators, public health authorities, academia, and industry stakeholders. Measures such as mandatory child-resistant packaging for cannabinoid-containing confectionery products and standardized testing protocols for cannabinoid transformations during food processing could improve consumer protection, market transparency, and product traceability while supporting responsible innovation. Such efforts may help address the interconnected challenges represented by the “CBD Triangle”: CBD’s pharmaceutical attributes, its regulatory status as a novel food, and persistent food safety concerns, including THC contamination, labeling deficiencies, and traceability gaps.

## Figures and Tables

**Table 1:** Distribution of CBD-related notifications by notification classification, risk decision, and food category across the years (RASFF, 2018–2025), presented as a heat map (with notification frequency increasing from green to red).

Year	2018	2019	2020	2021	2022	2023	2024	2025	Total
Number of Notifications	2	65	57	49	45	77	55	77	427
Classification									
alert	1	12	15	11	6	13	8	17	83
border rejection		1			1	2	1	1	6
information	1	52	42	38	38	62	46	59	338
Risk decision									
not serious		7	4			1		1	13
potential risk						37	35	54	126
potentially serious				1		19	12	9	41
serious	1	12	15	12	9	13	8	13	83
undecided	1	46	38	36	36	7			164
Food category									
alcoholic beverages						1		1	2
cereals and bakery products	1						1	1	3
cocoa, cocoa preparations, coffee, tea		5	5	3		3	2	6	24
confectionery		2	2		1	8	12	29	54
dietetic foods, supplements, fortified foods	1	52	43	43	34	52	36	24	285
fats and oils		2		1		1	1	6	11
food additives and flavourings					1	1			2
honey and royal jelly		2				2			4
ices and desserts					1				1
non-alcoholic beverages		1	2		2	3		1	9
nuts, nut products and seeds			1						1
other food product/mixed		1	4	2	6	6	3	9	31

Food category “dietetic food, supplement, fortified food” shows a major drop in notifications in 2025, while “confectionery” spikes dramatically. The product-level classification (see Section 2.3) indicates that newer notifications related to gummies were mainly categorized as “confectionery”, in contrast to the previously dominant classification under “dietetic food, supplement, fortified food”, reflecting differences in the interpretation of regulatory classification of such products. An additional reason for the observed spike in “confectionery” can be the potential enforcement of targeted sampling of gummies.

**Table 2:** A summary of CBD and THC concentrations in hemp/cannabis-based food, and related oral consumer products outside the RASFF system (since 2020).

Product Type/Matrix	No. of Samples	Cannabinoid(s) Reported	CBD Range	THC Range	Units *	Main Notes	Country/Market	Year Public.	Ref.
**Food supplements**	10	CBD, THC	12.87–51.57	ND	mg/mL	CBD supplements. Two correctly labelled, two under-8 over-labelled; all THC-free.	Hungary	2026	[28]
	131	THC	NR	max 3400	mg/kg	Hemp supplements; 98 THC positive.	Germany	2022	[29]
**CBD oils**	23	CBD	2.25–19.7	0.014–0.165	% *v*/*v*	CBD detected in all. Ten accurately matched the labelled conc. (±10%), 11 varied 3–25% from label value, 2 lacked CBD label claims. THC detected in 6, all < regulatory threshold (0.2%).	Malta	2026	[30]
	8	8, incl. CBD(A), THC(A)	0.94–20.87	-	%	3 appropriately labeled, 3 under-2 over-labeled. All met legal limit for THC/THCA.	Germany	2024	[10]
	10	9, incl. CBD, THC	ND-16.13	ND	%	3 over-labelled, 7 lacked CBD label claims; 2 labelled <0.2% THC.	Portugal	2024	[31]
	12	CBD	19.58–54.09	-	mg/mL	CBD mean cocnc. Of 35.51 mg/mL. Six appropriately labeled, one under-5 over-labelled.	Online (Hungary)	2023	[32]
	11	CBD, THC, CBN	NR	0.036–0.200	% *w*/*v*	Mean CBD vs label claim 91.56%; 4 appropriately labelled, 4 under-1 over-labelled, 2 lacked CBD label claims. THC detected in 6.	United States	2022	[33]
	293	THC	NR	NR	-	Hemp foods, mostly CBD oils. In total, 28 associated with Δ^9^-THC exposure > LOAEL (2.5 mg/day), 131 between ARfD and LOAEL, and 134 < ARfD.	Germany	2023	[22]
	80	THC	NR	0.008–2.071	mg/mL	Hemp-derived CBD oil products. Δ^9^-THC detected in 52 (51 brands, online and local retailers; mean 0.620 mg/mL). In total, 5 out of 21 labelled as “THC-Free” contained Δ^9^-THC (0.015–0.656 mg/mL).	United States	2022	[34]
	18	CBD, THC	16.12–242.6	ND	μg/mL	8 within acceptance limits for CBD content. Δ^9^-THC not detected—all compliant with EU legislation.	Belgium	2021	[35]
	2	CBD	9.2–18.9	-	mg/mL	-	Online	2020	[36]
	1	15, incl. CBD, Δ^9^-THC, THCA	45029	683	mg/kg	In relatively good agreement with declared values: ~4% CBD and <0.05% Δ^9^-THC.	Switzerland	2020	[37]
**Hemp seed oils**	5	CBD, THC	ND	ND	mg/mL	CBD-free and THC-free.	Hungary	2026	[28]
	20	CBD, Δ^9^-THC, THCA, Δ^8^-THC, CBN	0.11–24.34	0.11–31.08	µg/mL	CBD—strong variability; all except 1 had detectable THC. Laws in Türkiye: “zero tolerance” for Δ^9^-THC. CBN conc. of 0.02–3.81 μg/mL.	Türkiye	2026	[38]
	43	47, incl. CBD, Δ^9^-THC	0.47–156.3	ND-22	mg/kg	30 cannabinoids quantified in at least 1 sample. CBD in 29. Δ^9^-THC in 8; 70 kg individual consuming the recommended serving size of 14 g of hemp oil would exceed ARfD by consuming 3 samples. Ten labelled as “THC-free”: 0.44–13 mg/kg THC eq.	Europe (Czech Republic)	2025	[39]
	30	16, incl. CBD, Δ^9^-THC, THCA	0.11–6.68	0.2–6.7	mg/kg	3 > EU Δ^9^-THC eq. max. level for hemp seed oils (7.5 mg/kg).	Germany (Czech R., Austria, Lithuania)	2024	[40]
	9	CBD, THC	ND-31.7	0.4–22.5	mg/kg	3 > THC cutoff.	South Korea	2023	[41]
	137	THC	NR	max 233	mg/kg	102 THC positive.	Germany	2022	[29]
	17	CBD, total THC, CBN	NR	NR	mg/kg	THC as total THC.	Europe	2021	[42]
	7	CBD	ND-20.5	-	mg/mL	CBD detected in 6.	Online	2020	[36]
	4	15, incl. CBD, Δ^9^-THC, THCA	ND-15.5	ND-9.96	mg/kg	Very different cannabinoid profiles and levels.	Switzerland	2020	[37]
	11	CBD, THC, CBN	6.66–63.40	0.30–19.73	mg/mL	1 > national THC cutoff.	South Korea	2020	[43]
**Hemp seeds**	12	CBD, Δ^9^-THC, THCA, Δ^8^-THC, CBN	0.13–2.40	0.11–2.08	µg/g	CBD and Δ^9^-THC in most seeds. Laws in Türkiye: “zero tolerance” for Δ^9^-THC. CBN conc. of 0.05–0.21 μg/g.	Türkiye	2026	[38]
	30	47, incl. CBD, Δ^9^-THC	0.10–27.01	ND-3.1	mg/kg	21 cannabinoids quantified in at least 1 sample. CBD predominant in 25. Δ^9^-THC predominant in 5; 1 > legal threshold. A 70 kg individual consuming the recommended serving size of 40 g of hemp seed would exceed ARfD by consuming 1 sample. Among 10 labeled as “THC-free”, 1 without THC.	Europe (Czech Republic)	2025	[39]
	1	CBD, Δ^9^-THC, CBG, others	1.95	0.654	µg/g	-	Cyprus market	2023	[44]
	4	THC, CBD	ND-2.1	ND-4.2	mg/kg	Compliant with national standard.	South Korea	2023	[41]
	93	THC	NR	max 196	mg/kg	96% THC positive.	Germany	2022	[29]
	10	CBD, total THC, CBN	max. 72	ND–3.4	mg/kg	THC as total THC.	Europe	2021	[42]
	3	15, incl. CBD, Δ^9^-THC, THCA	0.49–1.06	ND-0.21	mg/kg	Very different cannabinoid profiles and levels.	Switzerland	2020	[37]
	77	CBD, THC, CBN	0.32–25.55	0.06–5.91	mg/g	1 (Australian) > national THC cutoff.	South Korea	2020	[43]
**Hemp protein**	6	CBD, total THC, CBN	ND-11	1.3–2.2	mg/kg	CBD NQ in 5. THC as total THC.	Europe	2021	[42]
	3	15, incl. CBD, Δ^9^-THC, THCA	ND-4.97	0.20–0.38	mg/kg	Products can have very different cannabinoid profiles and levels.	Switzerland	2020	[37]
**Hemp flour**	13	47, incl. CBD, Δ^9^-THC	0.24–23.15	ND-5.1	mg/kg	27 cannabinoids quantified in at least 1 sample. CBD predominant in 12. Δ^9^-THC predominant in 1, >legal threshold. THC quantified in all 3 labelled as “THC-free”.	Europe (Czech Republic)	2025	[39]
	7	9, incl. CBD, Δ^9^-THC, THCA	1.48–14.7	0.021–0.428	mg/kg	100% CBD positive. 100% THC positive.	Italy	2021	[19]
**Pasta (20% hemp flour)**	33	9, incl. CBD, Δ^9^-THC, THCA	0.038–2.70	0.020–0.140	mg/kg	77% CBD positive. 37% THC positive.			
**Bakery products**	22	9, incl. CBD, Δ^9^-THC, THCA	0.039–47.1	0.030–8.87	mg/kg	86% CBD positive. 55% THC positive.			
**Edibles**	26	CBD, THC	0.0–8.80	0.0–28.40	wt %	14 high CBD. THC in most; 12 high THC.	South Africa	2021	[20]
**Processed foods**	3	CBD, CBD	ND-3.1	ND-1.7	mg/kg	Compliant with national standard.	South Korea	2023	[41]
**Confectionery**	8	8, incl. CBD(A), THC(A)	ND-4.95	-	mg	CBD candies, chewing gums, gummy bears, cookies. In total, 3 appropriately labelled, 1 over-labelled, 4 lacked CBD label claims. All met legal limit for THC/THCA.	Germany	2024	[10]
	2	CBD, Δ^9^-THC, CBG, others	0.502–0.520	1.64–4.92	µg/g	Chocolate.	Cyprus market	2023	[44]
	7	CBD, total THC, CBN	NR	NR	mg/kg, mg/L	Includes chocolate-type products, lollipops.	Europe	2021	[42]
	3	15, incl. CBD, Δ^9^-THC, THCA	ND-0.43	ND	mg/kg	Chocolate. Products can have very different cannabinoid profiles and levels.	Switzerland	2020	[37]
**Hemp tea/infusion**	5	8, incl. CBD(A), THC(A)	ND-1.28	-	%	Plant material. In total, 2 under-2 over-labelled, 1 lacked CBD label claims. All met legal limit for THC/THCA.	Germany	2024	[10]
	6	9, incl. CBD, THC	0.01–0.26	0.001–0.01	%	Labels did not describe the conc. other than <0.2% THC.	Portugal	2024	[31]
	1	CBD, Δ^9^-THC, CBG, others	4381	180	µg/g	-	Cyprus market	2023	[44]
	89	THC	NR	max 588	mg/kg	88% THC positive.	Germany	2022	[29]
	6	CBD, total THC, CBN	NR	NR	mg/kg	Dried leaves + flowering tops included as hemp tea products.	Europe	2021	[42]
	129	CBD, THC	0.0–19.36	0.0–38.41	wt %	Infusion. In total, 43 high-CBD. THC in most; 86 high-THC.	South Africa	2021	[20]
	100	CBD, THC	0.0–13.64	0.0–30.88	wt %	Plant material. In total, 36 high-CBD. THC in most; 64 high-THC.			
	2	15, incl. CBD, Δ^9^-THC, THCA	1268–1348	11.2–12.9	mg/kg	Very different cannabinoid profiles and levels.	Switzerland	2020	[37]
**Coffee/mix for infusion**	1	CBD, Δ^9^-THC, CBG, others	0.042	ND	µg/g	Δ9-THC not detected.	Cyprus market	2023	[44]
	9	9, incl. CBD, Δ^9^-THC, THCA	54.2–1172.3	3.70–131.4	mg/kg	67% CBD positive. 67% THC positive.	Italy	2021	[19]
	2	15, incl. CBD, Δ^9^-THC, THCA	63.9–223	4.3–34	mg/kg	Very different cannabinoid profiles and levels.	Switzerland	2020	[37]
**Alcoholic and nonalcoholic beverages**	6	8, incl. CBD(A), THC(A)	ND-55.1	-	mg	Beer, liqueur, energy drinks, nonalcoholic drinks. In total, 2 over-labelled, 4 lacked CBD label claims. All met legal limit for THC/THCA.	Germany	2024	[10]
	6	9, incl. CBD, THC	ND	ND	-	3 beers, 2 ice teas, 1 carbonated beverage—no cannabinoids detected. Labels did not describe the presence of *Cannabis sativa* extract; packaging’s imagery—word cannabis + allusive images.	Portugal	2024	[31]
	1	CBD, Δ^9^-THC, CBG, others	0.830	ND	ng/g	Beer. Δ^9^-THC not detected.	Cyprus market	2023	[44]
	1	CBD, Δ^9^-THC, CBG, others	0.150	ND	ng/g	Energy drink. Δ^9^-THC not detected.			
	21	CBD, THC, CBN	NR *	0.0002–0.0005	% *w*/*v*	Mean CBD conc. vs label claim 59.93%. In total, 1 appropriately labelled, 2 under-11 over-labelled, 7 lacked CBD label claims. THC detected in 5, CBN in 2 (0.0015% *w*/*v*).	United States	2022	[33]
	61	THC	NR	max 0.53	mg/kg	76% THC positive.	Germany	2022	[29]
	14	9, incl. CBD, Δ^9^-THC, THCA	0.020–48.3	0.014–1.48	mg/L	93% CBD positive. 64% THC positive.	Italy	2021	[19]
	172	CBD, THC	0.0–50.38	0.0–84.49	wt %	84 high CBD. THC in most; 88 high THC.	South Africa	2021	[20]
	1	15, incl. CBD, Δ^9^-THC, THCA	0.0095	ND	mg/kg	Raw milk from cows fed with hemp feed.	Switzerland		[37]
**Extract**	398	CBD, THC	0.0–100.00	0.0–92.84	wt %	40 high CBD. THC in most; 336 high THC.	South Africa	2021	[20]
**Other food/oral consumer products**	7	CBD, THC, CBN	NR *	0.0008–0.0046	% *w*/*w*	Incl. chocolate bars. Mean CBD conc. vs label claim 67.01%. In total, 1 appropriately labelled, 2 over-labeled, 4 lacked CBD label claims. THC detected in 5, CBN in 1 (0.0001% *w*/*w*).	United States	2022	[33]
	7	CBD, THC	0.0–0.48	0.0–0.22	wt %	5 high CBD. THC in most; 2 high THC.	South Africa	2021	[20]
	8	CBD, THC	0.0–99.41	0.0–74.18	wt %	Other solid products. Four high in CBD. THC in most; 4 high in THC.			
	5	9, incl. CBD, Δ^9^-THC, THCA	0.040–1.64	0.036–0.311	mg/kg	80% CBD positive. 100% THC positive.	Italy	2021	[19]

ND = not detected; NR = not reported; NQ = not quantified; LOAEL = lowest observed adverse effect level; ARfD = acute reference dose. Units are reported as in the original publications and are therefore not directly comparable across matrices. Reference [33] reports CBD mainly relative to the label claim rather than as a single absolute concentration range. * The observed heterogeneity in measurement units reflects a broader issue within the field and highlights the need for greater standardization in future research and product reporting. Harmonizing the reported measurement units would greatly improve comparability among commercial products.

**Figure A2:**
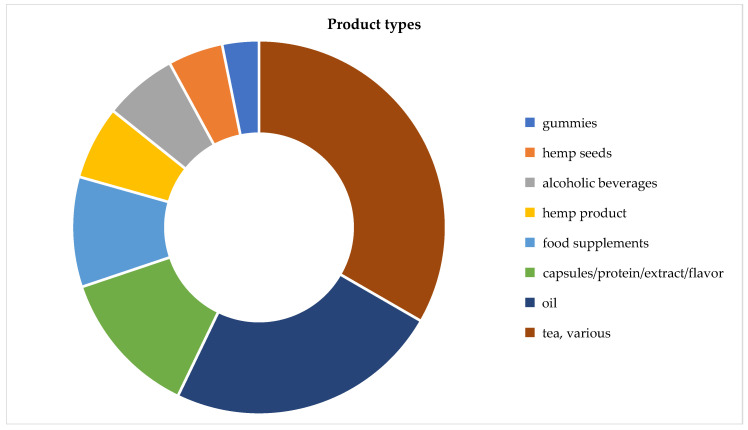
Distribution of hemp/cannabis-related notifications (CBD notification excluded) by product types (RASFF, 2018–2025).

**Figure A3:**
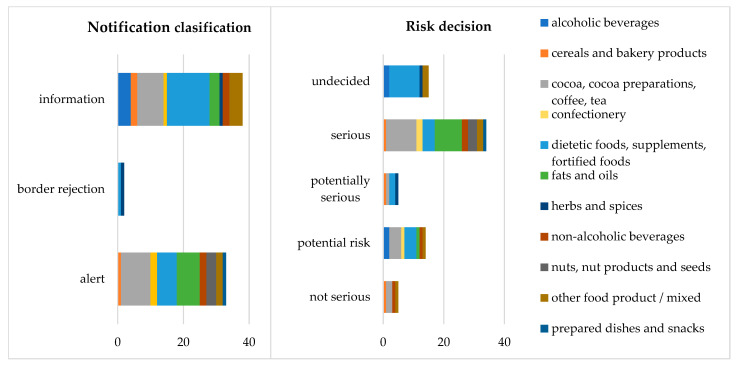
Distribution of hemp/cannabis-related notifications (CBD notification excluded) by notification classification and risk decision across food categories (RASFF, 2018–2025).

**Figure A4:**
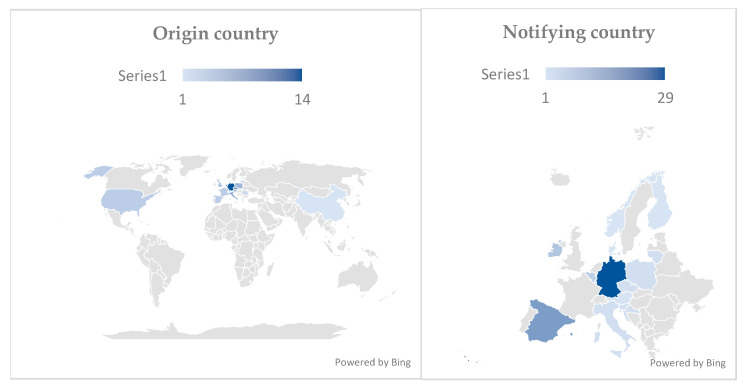
Geographical distribution of hemp/cannabis-related notifications by country of origin and notifying country (RASFF, 2018–2025) (Map generated using Microsoft^®^ Excel^®^ Powered by Bing™. Contains data from GeoNames, Microsoft, Open Places, and OpenStreetMap).

## Data Availability

Data available in a publicly accessible repository https://webgate.ec.europa.eu/rasff-window/screen/search (accessed on 3 January 2026).

## Supplementary Materials

The following supporting information can be downloaded at https://www.mdpi.com/article/10.3390/molecules31132287/s1, Table S1: Distribution of CBD-related notifications by product types (RASFF, 2018–2025); Table S2: Distribution of CBD co-reporting with THC and other cannabinoids (RASFF, 2018–2025); Table S3: Distribution of CBD-related notifications by notification classification across food categories (RASFF, 2018–2025); Table S4 Distribution of CBD-related notifications by risk decision across food categories (RASFF, 2018–2025).

## Abbreviations

The following abbreviations are used in this manuscript:

10-OH-HHC	10-Hydroxy-Hexahydrocannabinol
ALP	Alkaline phosphatase
ALT	Alanine transaminase
AST	Aspartate aminotransferase
CBC	Cannabichromene
CBD	Cannabidiol
CBDA	Cannabidiolic acid
CBG	Cannabigerol
CBN	Cannabinol
CYP	Cytochrome P450
EC	European Commission
EFSA	European Food Safety Authority
EU	European Union
GGT	Gamma-glutamyl transferase
H_4_CBD	Hexahydrocannabidiol
HHC	Hexahydrocannabinol
RASFF	Rapid Alert System for Feed and Food
THC	Tetrahydrocannabinol
THCA	Tetrahydrocannabinolic acid
THCP	Tetrahydrocannabiphorol
THCV	Tetrahydrocannabivarin
UGT	Glucuronosyltransferase

## Appendix A

**Table A1:** Chemical structures of cannabinoids and cannabinoid derivatives.

Name	Structure	Name	Structure
CBD Cannabidiol		CBDA Cannabidiolic acid	
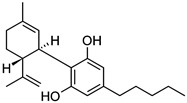
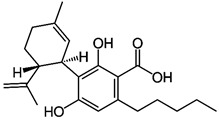
Δ^9^-THC/THC Δ^9^-Tetrahydrocannabinol		THCA Δ^9^-Tetrahydrocannabinolic acid	
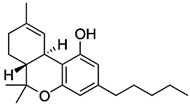
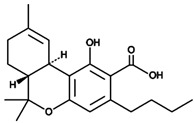
Δ8-THC Δ^8^-Tetrahydrocannabinol		CBN Cannabinol	
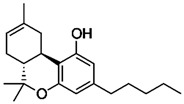
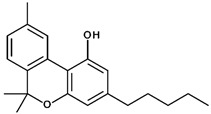
CBG Cannabigerol		CBC Cannabichromene	
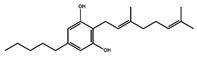
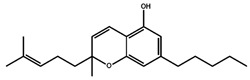
THCV Tetrahydrocannabivarin		THCP Tetrahydrocannabiphorol	
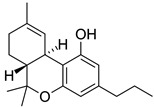
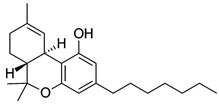
HHC Hexahydrocannabinol		10-OH-HHC 10-Hydroxyhexahydrocannabinol	
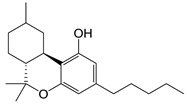
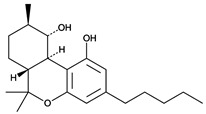
H4CBD Hexahydrocannabidiol (hydrogenated CBD)			
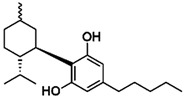

## Appendix B

**Table A2:** Distribution of hemp/cannabis-related notifications (CBD notification excluded) by notification classification, risk decision, and food category across the years (RASFF, 2018–2025), presented as a heat map (with notification frequency increasing from green to red).

Year	2018	2019	2020	2021	2022	2023	2024	2025	Total
Number of Notifications	2	11	9	12	10	9	8	12	73
Classification									
alert	2	4	5	8	6	3	1	4	33
border rejection	0	0	1	0	0	0	0	1	2
information	0	7	3	4	4	6	7	7	38
Risk decision									
not serious	0	1	1	1	0	0	0	0	3
potential risk	0	0	0	0	0	3	7	4	14
potentially serious	0	0	0	0	0	2	0	3	5
serious	2	4	5	8	6	3	1	5	34
undecided	0	6	3	3	4	1	0	0	17
Food category									
alcoholic beverages	0	0	1	0	1	0	1	1	4
cereals and bakery products	0	2	0	0	0	0	0	1	3
confectionery	0	0	0	1	0	0	1	1	3
cocoa, cocoa preparations, coffee, tea	0	2	1	3	3	2	2	4	17
dietetic foods, supplements, fortified foods	0	3	4	3	4	5	1	0	20
fats and oils	0	0	2	2	0	1	1	4	10
herbs and spices	0	1	0	0	0	0	0	1	2
non-alcoholic beverages	0	0	0	3	0	0	1	0	4
nuts, nut products and seeds	1	0	0	0	2	0	0	0	3
other food product/mixed	0	3	1	0	0	1	1	0	6
prepared dishes and snacks	1	0	0	0	0	0	0	0	1
