# Supplementary material for: Cannabidiol in Food and Food Supplements: Drug, Novel Food and Hazard Triangle
Source: Molecules. 2026 Jul 1;31(13):2287. doi: 10.3390/molecules31132287 (PMC13362604; doi:10.3390/molecules31132287)
Supplement: Supplementary file 1 [file molecules-31-02287-s001.zip › molecules-4375241-supplementary.pdf]

SUPPLEMENTARY MATERIAL

Cannabidiol in Food and Food Supplements: Drug, Novel Food and Hazard Triangle

Ljilja Torović [1,2,*], Katarina Urumović [1,2], Dunja Kobiljski [3] and Branislava Srđenović Čonić [1,2]

[1] Department of Pharmacy, Faculty of Medicine, University of Novi Sad, Hajduk Veljkova 3, 21000 Novi Sad, Serbia; katarina.bijelic@mf.uns.ac.rs (K.U.); branislava.srdjenovic-conic@mf.uns.ac.rs (B.S.Č.)
[2] Center for Medical and Pharmaceutical Investigations, Faculty of Medicine, University of Novi Sad, Hajduk Veljkova 3, 21000 Novi Sad, Serbia
[3] Department of Industrial Engineering and Management, Faculty of Technical Sciences, University of Novi Sad, Trg Dositeja Obradovića 6, 21000 Novi Sad, Serbia; dunjakobiljski@uns.ac.rs
* Correspondence: ljilja.torovic@mf.uns.ac.rs

	Content	Page
**Table S1**	Distribution of CBD-related notifications by product types (RASFF, 2018-2025)	2
**Table S2**	Distribution of CBD co-reporting with THC and other cannabinoids (RASFF, 2018-2025)	3
**Table S3**	Distribution of CBD-related notifications by notification classification across food categories (RASFF, 2018-2025)	3
**Table S4**	Distribution of CBD-related notifications by risk decision across food categories (RASFF, 2018-2025)	4

Table S1. Distribution of CBD-related notifications by product types (RASFF, 2018-2025)

**PRODUCTS**	**N**
biscuits	1
bonbons	1
CBD + cannabinoids	1
crystals	1
distillates	1
ice cream	1
alcoholic beverages (1 rhum)	2
jellies	2
lollypops	3
pasta	3
syrup	3
water	3
coffee	4
cookies	5
honey	5
oral spray	5
confectionery	5
candies	7
hemp extract/powder	7
non-alcoholic drinks	7
non-defined foods	11
non-defined CBD	13
tea and herbal infusion	13
chewing gums	14
chocolate sweets	15
CBD capsules	19
gummies	33
(CBD/cannabis/hemp) products	39
food supplements	81
(CBD/cannabis/hemp) oil/oil drops	150

Table S2. Distribution of CBD co-reporting with THC and other cannabinoids (RASFF, 2018-2025)

Combination of cannabinoids	N
**CBD + 10-OH-HHC**	1
**CBD + CBN**	1
**CBD + CBN + CBC**	1
**CBD + THC + HCC**	1
**CBD + H4CBD + THC**	1
**CBD + H4CBD + THCV + THCP + THC**	1
**CBD + THC + hemp extract**	1
**CBD + THCP**	2
**CBD + CBDA + THC**	2
**CBD + cannabinoids**	2
**CBD + CBG**	4
**CBD oils "full spectrum"**	4
**CBD + hemp/cannabis**	44
**CBD + THC**	108

Table S3. Distribution of CBD-related notifications by notification classification across food categories (RASFF, 2018-2025)

Food category	Notification classification			
	alert	border rejection	information	**Total**
**alcoholic beverages**			2	2
**cereals, bakery products**			3	3
**cocoa, cocoa preparations, coffee, tea**	4	1	19	24
**confectionery**	14		40	54
**dietetic foods, supplements, fortified foods**	59	5	221	285
**fats and oils**	2		9	11
**food additives, flavourings**			2	2
**honey, royal jelly**	1		3	4
**ices and desserts**			1	1
**non-alcoholic beverages**	1		8	9
**nuts, nut products, seeds**			1	1
**other food product / mixed**	2		29	31
**Total**	83	6	338	427

Table S4. Distribution of CBD-related notifications by risk decision across food categories (RASFF, 2018-2025)

Food category	Risk decision					
	not serious	potential risk	potentially serious	serious	undecided	**Total**
**alcoholic beverages**		2				2
**cereals, bakery products**		2			1	3
**cocoa, cocoa preparations, coffee, tea**	1	9		6	8	24
**confectionery**	1	27	11	12	3	54
**dietetic foods, supplements, fortified foods**	9	61	27	59	129	285
**fats and oils**		7		2	2	11
**food additives, flavourings**					2	2
**honey, royal jelly**			2	1	1	4
**ices and desserts**					1	1
**non-alcoholic beverages**	1	1	1	1	5	9
**nuts, nut products, seeds**					1	1
**other food product / mixed**	1	17		2	11	31
**Total**	13	126	41	83	164	427
